# A multi-source behavioral and physiological recording system for cognitive assessment

**DOI:** 10.1038/s41598-023-35289-z

**Published:** 2023-05-19

**Authors:** Zi-yang Wang, Li Liu, Yu Liu

**Affiliations:** State Key Laboratory of Multimodal Artificial Intelligence Systems, The Institute of Automation, Chinese Academy of Sciences, Beijing, China

**Keywords:** Biomedical engineering, Electrical and electronic engineering, Psychology

## Abstract

Cognitive assessment has a broad application prospect, including estimate of childhood neuro development and maturation, diagnosis of neurodegenerative diseases, and selection for special profession. With the development of computer technique and behavioral recording sensors, the method of cognitive assessment has been replaced from paper scale test to human–computer interaction. We can not only obtain the results of tasks, but also make it possible to acquire multiple behavioral and physiological data during the task. However, there is still a strong challenge of recording multi-source data synchronously during multi-dimensional cognitive assessments. Therefore, we built a multi-source cognitive assessment system can record multi-pattern behavioral and physiological data and feedback at different spatiotemporal levels. Under this system, we developed a multi-source diagnostic toolset for cognitive assessment, including eye tracking, hand movement, EEG and human–computer interaction data during the cognitive task. 238 participants with different mental disorders were assessed using this system. The results showed that our diagnostic toolset can be used to study the behavioral abnormalities of patients with mental disorders through the characteristics of multi-source data. Furthermore, this system can provide some objective diagnostic criteria such as behavioral characters and EEG features for diagnosis of mental disorders.

## Introduction

Cognitive assessment is using a sort of tests, tasks and paradigms designed to identify fundamental features of human cognitive skills. Actually, the theory and method of assessing the cognitive abilities receive a consistent controversy for more than 100 years^1^. From a single dimension of intelligence assessment, gradually developed into a multi-dimensional cognitive abilities’ assessment. There have also been several iterations of theoretical approaches, including the Spearman’s g intelligence theory, fluid and crystallized (Gf and Gc) intelligence theory, etc.^2^. According to the research of cognitive abilities hierarchical structure, the general result of first-order abilities consist: Visualization and spatial orientation abilities (Gv), Abilities of listening and bearing (Ga), Acculturational knowledge abilities (Gc), Abilities of reasoning under novel conditions (Gf), Abilities of short-term apprehension and retrieval (SAR), Abilities of long-term storage and retrieval (TSR), Speed of thinking abilities (Gs) and Quantitative mathematical abilities (Gq)^3–5^. Although the application of cognitive assessment has extended nowadays to personnel selection and training, early childhood education, children’s education, giftedness identification, etc.^6^. the most important usage of cognitive assessing method is to explore cognitive abilities bias of persons that involved different mental disorders^7,8^.

Early cognitive assessments are mostly based on scales or direct interaction between the experimenters and the subjects. The scale is similar to an exam. It will ask the subjects to answer some specific questions, such as some mathematical questions, semantic understanding, vocabulary examination, logical reasoning, etc. Some representative scales in intelligence assessment are Wechsler Adult Intelligence Scale, Stanford-Binet Intelligence Scale and Differential Ability Scales. For cognitive test tools, such as the Wisconsin Card Task, which examines cognitive flexibility, also used actual interaction to examine the cognitive ability of subjects in the early stages. Nowadays, due to the development of computer interaction technology and sensor technology, cognitive ability assessment based on computer platforms has gradually replaced the traditional assessment methods, and it also become possible to record a variety of behavioral and physiological data during the cognitive task.

Because the mental disorders do not only cause specific behavioral performance, it also led to some significant changes of the cognitive abilities. Therefore, Cognitive ability assessment has also been used in the research of many kinds of mental disorders, including Neurocognitive disorders (NCD), neurodevelopmental disorders, depressive disorders, anxiety disorders, etc. especially in the children’s development, such as Autism spectrum disorder (ASD), Attention-Deficit/Hyperactivity Disorder (ADHD) and sensory disorders. Actually, according to the Diagnostic and statistical manual of mental disorders (DSM-5th ed.), nearly all of the mental disorder has cognitive abilities problem, and only disorders whose core features are cognitive are included in the NCD category. The NCDs are those in which impaired cognition has not been present since birth or very early life, and thus represents a decline from a previously attained level of functioning, such as Complex attention, Executive function, Learning and memory, Language, perceptual-motor, and Social cognition. Children with ASD have difficulty with attention, social interaction, and language understanding. ASD do not have overall strengths in nonverbal processing, and instead have strengths in visual perception. Behavioral difficulties are often signing of frustration and fatigue in individuals with ASD^9,10^. ADHD frequently occurs comorbid with other learning disorders (e.g., specific learning disabilities) and psychiatric diagnoses (e.g., oppositional defiant disorder, anxiety disorders). In attentive type of ADHD may be a neuropsychological and behavioral distinct disorder. It is also possible that some inattentive children may have ADHD but do not meet the behavioral threshold for hyperactive-impulsive symptoms; others may have other psychiatric of learning disorders. Despite convincing evidence that children with ADHD have significant cognitive, neuropsychological, academic, and behavioral problems due to frontal-subcortical circuit and executive dysfunction, it is common practice to focus largely on behavioral criteria for determining ADHD diagnosis and evaluating treatment effects^11,12^. Physical and sensory functioning have long been part of standard neuropsychological assessment. Both sensory and physical abilities are prerequisites for input and output mechanisms of cognitive faculties and provide indicators of the cognitive system’s integrity. The declines in sensory-perceptual abilities always linked with the decrease in a sensory modality or the damage of functions in neural system. Some behavioral deficits resulting from these disabilities clearly originate in biological function, such as lesions in the left frontal area of the brain may result in language deficits^13,14^.

Many psychiatric diseases can directly cause brain functional or mental disorders, but it is difficult to find any exact evidence of brain or functional brain lesions in the early stage of the disease. In this case, imaging examination only provide limited help for mental disorder diagnosis, so it is necessary to consider the correspondence between human behavior, mental state and disease^15^. For a long time, the diagnosis of diseases related to brain function is mainly based on the self-scoring of manuals or scales and the observation and empirical judgment of doctors, such as asking patients and their family members of medical history, using laboratory examinations and other methods to systematically assess the physical health status, and then, give a diagnosis of mental disorder^8^. However, this diagnosis method is obviously subjective and empirical, and the manifestations of mental symptoms or brain dysfunction symptoms often have crossover manifestations under different diseases. The traditional detection and diagnosis methods are relatively single, but for specific group of people whose suffered from specific disease, it is necessary to develop a multi-directional diagnosis. For example, Eye-tracking technology is an effective tool to study complex cognitive processes, such as using eye-tracking to measure cognitive load^16^, use eye-tracking to analyze the readability of LINQ code^17^. Real-time evaluation of attention state by EEG has been widely used in attention evaluation^18^, attention and meditation training, and driver fatigue driving, and has gradually become a golden-standard of attention evaluation. Although there are platforms that integrate a variety of cognitive abilities assessment paradigms, such as PsyToolkit^19^, Jacknaglieri^20^, BrainGymmer^21^, etc., which have developed a variety of online psychological and behavioral assessment or only for children’s cognitive ability assessing, however it is also necessary to provide behavioral data such as eye movements, hand movements and physiological data of mental disorders for auxiliary diagnosis.

In order to solve these problems, we need a novel system to computerize the cognitive test tools and access the behavioral and physiological test equipment, such as eye tracking, hand tracking, audio recorder, 3-lead EEG and olfactory sensors. The software of this system can record not only response time, task time and task score, but also the synchronized multi-pattern behavioral and physiological data for a universal cognitive assessment. The rest of this paper is organized as follows. “System architecture” section demonstrated the system structure and the design of every module in detail. “Cognitive assessment paradigm” section illustrates the behavioral paradigms of cognitive assessment used in our test-bed. “Result and discussion” section presents the experimental results and discusses the multi-pattern behavioral and physiological data of this system. Finally, in “Conclusions” section, conclusions are drawn and future work is outlined.

## System architecture

In order to increase the applicability and convenience for users, we built a web-based online cognitive assessment version and a client-based cognitive assessment version of the system. The client-based system is mainly for users who need to record not only task result data, but also eye tracking, EEG, and hand movement data, so it includes all external devices, such as EEG measurement module, motion sensors, and eye tracking module. The web-based online assessing system is designed for the users who only need human–computer interaction results of the tests. Therefore, the web-based online assessment system is not available to connect to external devices such as EEG, and can only acquire basic information such as the task results and response time of the subjects. In order to ensure the data interconnection efficiency between the system and a variety of external devices, the client system used the C language to construct the main frame of the system. Except for EEG experiment and hand movement test, the rest of assessments are all developed using HTML5, allowing users to assess various cognitive abilities on web pages. In the main client program, the corresponding HTML online cognitive assessing experiment page is obtained through HTTP request. The web-based system was built by using the Canvas element of HTML5, and used JavaScript to complete the image drawing, animation effects and task logic on the web page. In order to facilitate document operation, event handling and animation design, the jQuery framework was also used in the front end. We used a lightweight Nginx proxy server in the back end. The backend was developed with php language, and MySQL relational database was used. Since this system includes client-side development in C language and PHP development on the web side, the open-source NetBeans was selected to realize all web-based development.

**Figure 1:**
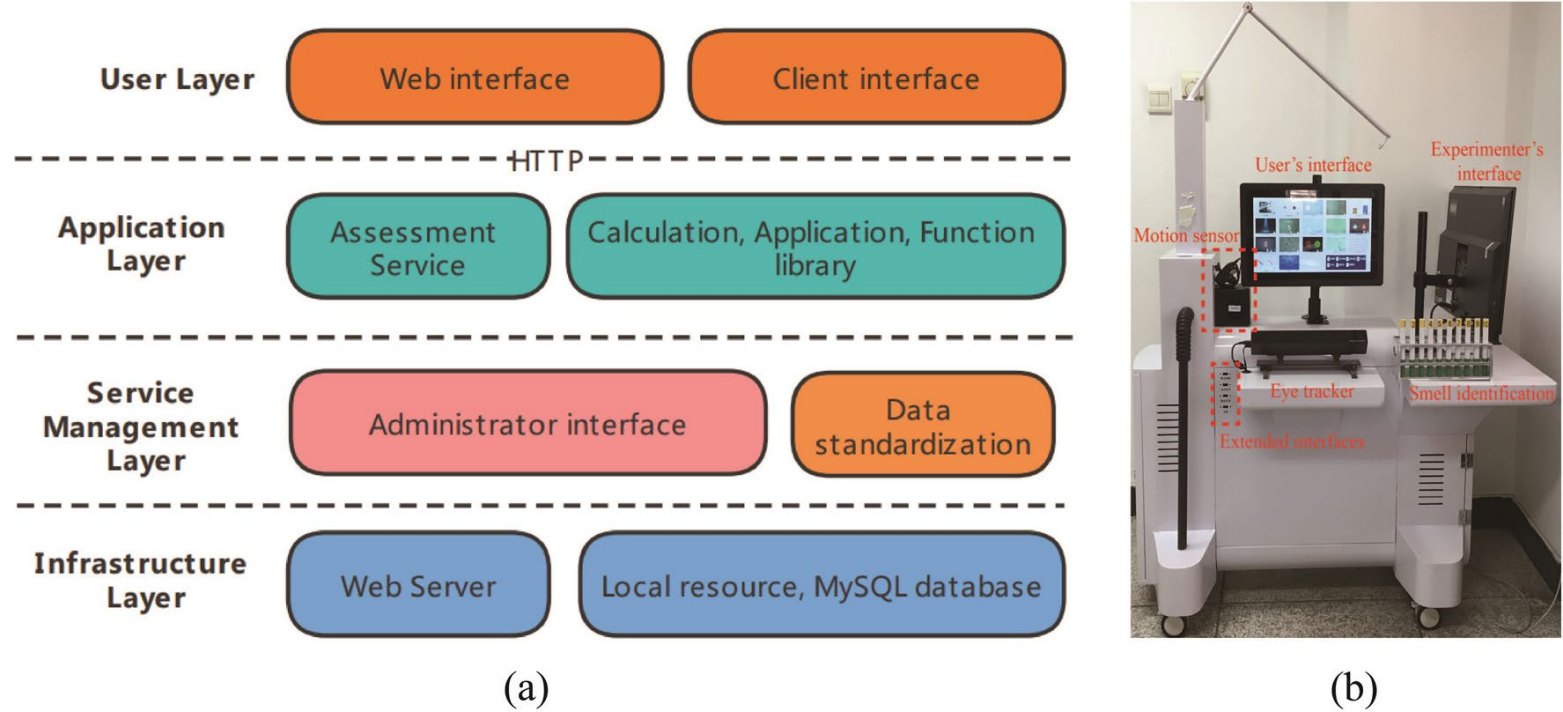
Overview of cognitive assessment test-bed. (**a**) Architecture of the system. (**b**) Cognitive assessment test-bed.

The overall structure of the system is shown in Fig. 1a, including User layer, Application layer, service management layer and infrastructure layer. In the user layer, we designed a client version and a web-based version. The client version of the system is shown in Fig. 1b. We designed an experimental console for this system. The patients perform cognitive assessment tasks in the user’s interface, and the experimenter can directly observe the patient's operation on the screen next to it. The peripheral modules in the system include eye tracker, scent pen, headset, motion tracker and EEG module. The web-based version can be used for 14 cognitive assessment tests such as cognitive control and memory test in any networked computer. However, because there is no peripheral module connected, it can only upload parameters such as behavior results and reaction time of the assessments to the server, and cannot obtain information such as EEG or eye tracking. In the application layer, we design the realization logic and result calculation method of each cognitive assessment. Due to the different types and sizes of the behavioral result data of the cognitive assessment and the other behavioral and physiological data files, different data processing interfaces have been designed. We have constructed a corresponding result data structure for each cognitive evaluation paradigm, and stored it in the MySQL database in the server. EEG raw data, motion movement data, eye movement data, etc. have been uploaded to the server in the form of files.

**Figure 2:**
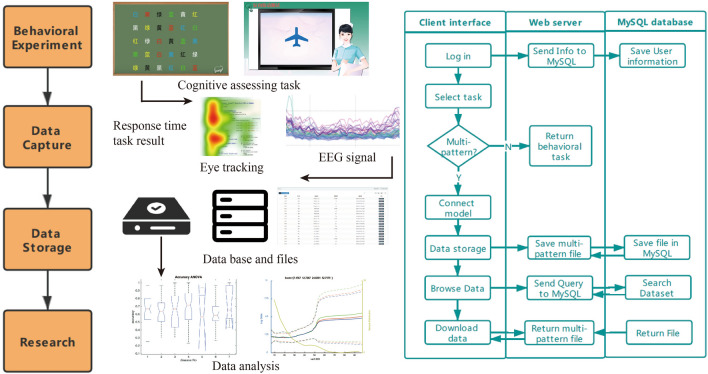
Workflow and interaction diagram among modules of system.

Workflow of the system is shown in Fig. 2. First, the subjects should register and log in the client or web-based version, and then select an experimental paradigm. If the selected test does not need to connect to the peripheral module, the client will send an http request to call the web page of the test, such as attention test, memory test, etc. If the selected test needs to connect peripheral modules (such as EEG, motion tracker and eye tracker), the experiment is performed in the client. After the experiment, the log file and original data will be uploaded to the server. The administrator can log in directly from the web page, and then filter or download experimental data based on the user’s information such as experiment date or user name. We have reserved multiple ports for both the client and the web terminal, which can accommodate 150 participants making cognitive assessment simultaneously.

At present, it takes approximately 60–90 min to complete all the tasks, 1–2 breaks are needed according to the status of patients. Here are some examples shown in Table 1, experiments such as attention and memory that are not involved in any peripheral sensors, we mainly record the trail number of the experiment, response time of every operation, and the accuracy or result of the task, etc. Taking memory test as an example, we recorded the difficulty and error of angle memory, the time used for each angle rotation, the original choice under the DNMS test, the response time of each operation in DNMS, the accuracy of DNMS, and time consumption and accuracy of long-term memory task.

**Table 1:** The information of experimental data (an example table).

Cognitive task	Data information
Attention task	Trail number, frame number, randomized cycle’s order, cycle’s color, number near the cycle, user’s color answer, user’s number answer
Memory task	Trail number of angle task, degree of difficulty, original angle, target number, target duration, button that user pressed, angle error, response time, picture’s number of DNMS, duration of each trial, target duration, target picture’s position, user’s answer, response time, LTM accuracy
Free-viewing task	Picture’s number, view distance, screen size, screen coordinates, pupil parameters
EEG recording	Static EEG original data, EEG under music stimuli
Hand-movement task	X axes, Y axes, Z axes, azimuth rotation, elevation rotation, roll rotation

If the experiment involves eye tracking to assess the cognitive abilities, we also obtained the eye tracking data synchronously in the test. For the EyeLink device (EyeLink 1000 Plus, SR Research Co., Ltd, Cannada), we store it in the server in EDF format, while in the EyeControl device (EyeControl, Shanghai Qingyan Technology Co., Ltd, Shanghai, China), we got the coordinate changes in txt format and add the marked point in the data. In the experimental paradigm that uses EEG data to analyze a variety of mental disorders, we have obtained 3-lead EEG data, and the EEG recording equipment is synchronized with the experimental program itself to obtain EEG data in the resting state and listening to music. After the experiment, the original data was uploaded to the server in the form of .csv. In the hand movement test task, we recorded the space axis data, the data of the azimuth angle, the elevation angle, and the roll angle during the task, and synchronized the experiment and the recorded data using different marks. After the test, the original data was stored in .txt format and uploaded to the server.

The parameters of peripheral module in the proposed system are shown in Table 2. Among them, the motion tracker uses a motion tracking module (Polhemus G4, Inc. dba Polhemus, Colchester, Vermont, USA) with 120 Hz sampling frequency, and it can record 6-degree of freedom motion tracking parameters, including X, Y, Z axes, Azimuth, Elevation and Roll. The EEG recorded using a portable 3-lead EEG instrument made by Hubin’s Lab in Lanzhou University, with a sampling rate of 250 Hz. According to the international 10–20 system, this module recorded Fp1, Fp2 and Fpz regions’ EEG using patch electrodes and the reference electrode is at A1 region. Eye tracker uses EyeLink 1000 instrument with 1000 Hz sampling rate, which can collect the X, Y coordinates and Pupil size on the screen. Red and green 3D glasses are used for the binocular rivalry test. 10 scent pens for smell identification test are provided by the Institute of Psychology, Chinese Academy of Sciences. Those smells are: cumin, rubber, cola, grape, vanilla, red dates, rose, apple, green tea, and walnut.

**Table 2:** Technical parameters of the system.

Technical parameters	Value
Motion tracker	120 Hz, X, Y, Z, A, E, R
Scent pen	10 kind of smells
EEG	3-lead, 250 Hz
Eye tracking	1000 Hz; X, Y and pupil size
3D glasses	Red/Green

## Cognitive assessment paradigm

According to the theoretical framework of cognitive ability assessment, consult the Diagnostic and statistical manual of mental disorders and the recommendations of psychiatric doctors. We screened 16 assessment tasks, which included all the factors in the first order of C-H-C cognitive assessment theory, and integrated 16 cognitive ability tools in this system. In fact, there are some differences of cognitive ability structure between the cognitive assessment theory and the neurocognitive disorder diagnosis method. For example, the diagnosis of neurocognitive disorder will focus on complex attention, including sustained attention, selective attention, etc. However, attention is not classified into a single category in the framework of normal cognitive assessment. The prime assessment structure in neurocognitive disorder contains Complex attention, executive function, learning and memory, language, perceptual-motor and social cognition. Although the cognitive assessment structure used to detect abnormal cognitive abilities has been relatively mature in the screening of mental disorders, we hope that the cognitive assessing provided in our system can effectively augment the existing cognitive structure. Therefore, we used a more general cognitive ability assessment structure to classify our 16 cognitive ability assessment methods, which illustrated that our test-bed can not only be used for cognitive assessment of patients with mental disorders, but also for other types of cognitive assessment after adjusting the test parameters, such as children's education, military selection, etc. The results are shown in Table 3. The interaction data contains the task accuracy, response time and the operation of the participants.

**Table 3:** Cognitive assessments’ classification.

Cognitive ability label	Cognitive assessment method in our system	Data recording
Visualization and spatial orientation abilities	Free-viewing test	Eye tracking
	Eye tracking test	Eye tracking, interaction data
	Biological motion recognition	interaction data
	Binocular rivalry	Alternative frequency and durations
Abilities of listening and hearing	Hearing test	Interaction data
Acculturational knowledge abilities	Language test	Interaction data
	Social attention	Interaction data
	Perception of risk	Interaction data
	Smell identification test	Interaction data
Abilities of reasoning under novel conditions	Anti saccade	Eye tracking
	Cognitive control	Interaction data
	Decision test	Interaction data
Abilities of short-term apprehension and retrieval	Attention test	Interaction data
	Memory test	Interaction data
Abilities of long-term storage and retrieval	Memory test	Interaction data
Extended test	EEG test	EEG raw data, probability of mental disorders
	Hand movement test	Interaction data, hand tracking

According to cognitive assessment theory, the participant’s response time (Gs) is also a very important parameter. The response time can often reflect the intelligence in normal people, and the details of behavior in different mental disorders. Therefore, our system recorded the specific response time and duration of the participant at each step of the operation. Hand movement test and EEG test are not classified into the existing categories of cognitive ability assessment, in fact hand movement is a sort of quantitative test of perception, which is focus on some specific behaviors. The result of hand movement also plays a very important role in mental disorder diagnosis, such as schizophrenia. EEG belongs to physiological data and can also be regarded as a comprehensive manifestation of a series of behaviors of pyramidal cells of cerebral cortex. There are many studies using EEG data to classify mental disorders, but the use of EEG to assess cognitive status only contains attention status, cognitive load, meditation, and other basic mental state. Therefore, we did not classify these two types of tests into the standard cognitive assessment theoretical framework, but these two behavioral and physiological data can also provide important evidences for the diagnosis of mental disorders or cognitive state research. The following is the brief description of 16 assessment methods:

### Free viewing test

Here we used Free-viewing paradigm^22,23^ to record the participant’s observation pattern. Two images are used in this test, one is human face, the other is landscape photo. Both display 15 s, and eye tracking information are recorded. In this test, Scan pattern of the participant could be acquired, and we may understand the processes that underlie the acquisition of vital visual information from the environment that is relevant to current tasks and goals. The result of this test is the participant’s eye tracking data and preferred gaze area.

### Eye tracking test

Saccade paradigm^24^ and smooth pursuit eye movement (SPEM) paradigm^25^ are used in this test. In the saccade paradigm, there will be a target point around the center of the screen, and the subject needs to keep staring at it. In some trials, interference point may appear randomly to the left, right, above, or below the center point. There are 15 trials in this task, and each trial takes 5 s. In the smooth pursuit eye movement task, the target point moves along the Lissajous-curve, and the target point only needs to complete the complete curve once. Visual attention control is a bottom-up goal-driven processing process, which can effectively reflect the visual processing ability. For example, the visual attention ability of ASD is complete or even enhanced in some aspects, but there are defects in other attention functions, and the schizophrenia also has symptoms of impaired eye movement^26^. The result of this test is to obtain the accuracy of the saccade area, the response speed, and whether it is disturbed.

### Biological motion recognition

Here we adopt an abstract biological movement recognition paradigm^27,28^, which assess the visual perception of motion patterns characteristic of human in locomotion. Biological movement perception is one of the most basic social adaptability. In this test we used abstract points as carriers of the motion, which was used as the stimulus material. In the experiment, we set up an upright walk to the left, an upright walk to the right, an inverted walk to the left, an inverted walk to a right, a total of four different stimulus materials. There are 64 random trials in this task with one second for each trial, each condition is repeated 16 times. Participants are required to judge the direction of light spot movement as accurately as possible. The result of the experiment is to obtain the task accuracy of subjects.

### Social attention

Dot probe paradigm and eye gaze paradigm are used in this test^29^. We have prepared 8 face pictures, 4 of which look to the left and 4 to the right, 4 are positive emotions and 4 are negative emotions. In the experiment, the light spot will appear on the left or right side of the picture for 50 ms, after the picture appears 500 ms. When the light spot disappears, the face picture still maintains 150 ms. There are 8 faces with light spots on both sides, so there are 16 trials, and then repeat 3 times for a total of 48 trials. This test is to evaluate the participant's attention preference to social stimuli, the characteristics of the individual's attention resources in the spatial allocation, and to examine the impact of the participant's positive and negative emotions. The result are calculated as Correct response (inconsistent conditions-number of consistent conditions)/(inconsistent conditions + number of consistent conditions) × 100%.

### Binocular rivalry test

This experiment used binocular rivalry paradigm^30,31^, which is that when the images presented by the corresponding positions of the retinas of the two eyes are inconsistent, our visual perception will randomly switch between the images of the two eyes. The stimuli were composite images of a red concentric ring (8 cycles/°) and a green radial grating (total 8 polar cycles in the pattern) with an average luminance of 135 cd/m^2^, sine wave modulated at a contrast level of 0.9, which was supported by the Institute of Biophysics, Chinese Academy of Sciences. Subjects viewed a composite of red circular and green radial gratings through a pair of red–green anaglyphic glasses. Participants need to click on the space after seeing the color change. This test will last 4 min. The result of this test is to record the time of appearance of the two images.

### Hearing test

Local–global paradigm and two-alternative forced choice paradigm are used in this task^32,33^. In the local–global test, we prepared 52 “di-di-di-di” and 20 “di-di-di-do” two voices, the subject have to distinguish the two voices and then answer the number of “di-di-di-do” in the end of the task. The sound is presented in a random order, but there will be no consecutive "di-di-di-do". The interval between the voice strings is random between 1.2 and 1.5 s, and varies from subject to subject. In the second test, In the second test, we still use 4 sounds to stimulate, but the first three sounds have the same frequency, and the frequency of the fourth sound will be higher or lower than the first three syllables. Participants need to choose whether the fourth voice is high or low frequency, there are 30 trials in this test. In this task we prepared 1189 Hz, 1365 Hz, 1384 Hz, 1443 Hz, 1463 Hz, 1681 Hz, six different voices to be distinguished for subjects. The result of the experiment is to obtain the memory accuracy of the difference sequence and the accuracy of sound recognition.

### Language test

In this test we used two tasks to assess the language ability. The first task is a word comprehension task. 4 words are displayed on the screen. One of the words has a different meaning from the other three. Participants need to choose words with different meanings. There are 16 trials in this test. The second task is to look at the picture and talk for 2 min. The result of this test we will record the accuracy of word comprehension and obtain the audio information of the participant.

### Anti saccade

This task is widely used in clinical research to assess the subject's ability to flexibly control and inhibit response^34^. Studies have shown that neurological diseases affect the function of the prefrontal lobe and basal ganglia, and the decline in this response ability is related to it. Inhibit the control field. The test has a total of 50 trials, 25 pro-saccade and 25 anti-saccade. The result of the test is to obtain the subject’s eye tracking and check the accuracy of the task.

### Perception of risk

Here we used inspection game to quantitatively calculate personal risk tolerance. Risk tolerance is a very important determinant in our decision-making process^35^. Risk tolerance is also related to some mental disorders, such as schizophrenia, anxiety disorders, substance addiction, and gambling addiction. In this test, we prepared two options for the participant. One option has a fixed award of 1, the other option has a 50% probability that the award is 2, and a 50% probability that there is no award. There are 15 trials in this test, and the result is to calculate the full award.

### Cognitive control test

Here we used Color-word matching Stroop task^36^ to assess the cognitive control ability. In this test, the participants are told to say the color of each word in the screen, not to be disturbed by the content of the word itself. There are 4 trails in this test, each trail contains 30 words that evenly arranged on the screen. In the first and the fourth trial, the meaning of words is not relevant to color, in the second and third trail, the meaning of words is relevant to color. The result is the time difference between interference reading and normal reading.

### Attention test

Attention test is designed from shape and number recognition problem, which is a visual attention feature binding task^37,38^. Parietal lobe is related to spatial attention, and plays an important role in feature binding. Under the condition of distracted attention or attention overload, the characteristics of the target and the distracting object are exchanged. In this task, the figure of the same color is on the left, the number is on the right of the shape element, and the numerals in the range of 1–9. In each trail, there are 24 frames (400 ms for each frame) and only one frame of the picture with a circular shape which is the target shape that the participant has to remember and then choose the color and number answer in the end of each trail. There are 15 trails in this test and the result is calculated as: color accuracy: (Correct quantity + 1/2 uncertain quantity)/total × 100; number accuracy: (First number × 3 + second number × 2 + third number × 1)/(total times × 3) × 100.

### Memory test

There are three different tasks in this memory test, including angle reproduction task, a long-term memory task, and the delayed non-match to sample (DNMS) paradigm^39,40^. In the angle reproduction task, there are two difficulties, and 2 trials of each difficulty, participants have to memorize 3 or 4 pencils’ angle which has different color and in different position on the screen. One of them will appear after 3 s delay, and participants need to adjust the pencil to the original angle in 10 s. The DNMS test is also divided into 2 levels of difficulty, each with 4 trials. Participants first observe the angle of 1 or 2 pencils for 1 s, then 2 or 4 landscape pictures appear on the screen for 2 s, and 3 or 5 landscape pictures appear on the screen, then participants need to select the one that did not appear before, then the target pencil appears on the screen after selection, and the subject needs to rotate the pencil to the original angle. In the long-term memory test, the participant first needs to memorize 20 animal pictures within 1 min. After the angle test is completed, the program presents 20 new animal pictures, and the participant needs to Find 10 pictures that appeared for the first time in these 20 pictures. The test result will calculate the angle memory error and the accuracy of long-term memory.

### Smell identification test

This test requires subjects to choose the name of what they smell from several options^41^. The olfactory processing is quite complicated and has high clinical value. This olfactory naming test is used to measure an individual’s ability to name odors, which is one of the indicators of physical and mental health. In this test, we prepared 10 scent pens provided by the Institute of Psychology, Chinese Academy of Sciences. We provided 4 options for each scent pen. Participants needed to choose the name of the smell they smelled. Participants also need to fill in a simple scale after completed the smell identification, including whether they smoke, drink alcohol, whether they suffer from respiratory allergies such as rhinitis, self-evaluation of smell, and medication consumption. The result of this test is the accuracy of olfactory recognition.

### Decision test

Similar to a general game task, this test uses a double-choice method to evaluate the ability of the subjects to find the best law of return^42^. In this test, there are two doors on the screen, and there is a yellow or red treasure chest behind each door. After the treasure chest is opened, a gold coin will be randomly obtained. The probability of this test is as follows: 80% of the probability behind the left door is a yellow treasure chest, a 20% probability is a red treasure chest, a yellow treasure chest has an 80% probability of getting gold coins, and a red treasure chest has a 20% probability of getting gold coins. Behind the right door, there is a 20% probability of being a yellow treasure chest, an 80% probability of being a red treasure chest, a yellow insurance has 80% probability of getting gold coins, and a red treasure chest has a 20% probability of getting gold coins. After trying a few times, the subject should find that it is relatively advantage to choose the left door. There are a total of 50 trials in the experiment. In 7–30 trials, there is a 10% probability that the probability of getting gold coin will be reversed (that is 80% for red treasure chest and 20% for yellow treasure chest), and each reversal will last for 3 trials. 31–40 trials will not get gold coins anyway, the purpose of this stage is to induce negative emotions, and after the 40th trial is completed, the subject will be asked whether to continue the task. If the subject chooses not to continue, it will be in advance end the task. This test mainly obtains the participants' cumulative income and choice after negative emotion induction.

### Hand movement test

In this test, we used the behavioral paradigm of tapping the screen. Participants need to wear a motion sensor on their hands, then raise their hands to tap the target point on the screen, and then lower their hands. There are 200 trials in this test. This paradigm can effectively evaluate Parkinson's disease, schizophrenia, etc. This test will obtain the participant’s tapping interval, tapping accuracy, and hand movement coordinate data.

### EEG test

The EEG experiment^43,44^ includes two stages: the first stage is the recoding of resting EEG with closed eyes, and the recording time is 90 s; the second stage is the EEG acquisition under audio stimulation, after the resting state EEG collection is completed, the participant rests for 1 min, and then waits for the EEG to stabilize, and then plays six audio segments to obtain the EEG signal under the subject’s audio stimulation for 72 s. The test detects mental disorders of the subjects by analyzing the power spectrum and other characteristics of EEG, and the system will finally give the subjects the probability of multiple mental disorders.

### Participants

Experiments was approved and executed by Shanghai Mental Health Center. During the whole experiment, we carried out relevant studies in strict accordance with the ethical rules of the Ethics Committee of Shanghai Mental Health Center. All methods were performed in accordance with the relevant guidelines and regulations. Informed consent was obtained from each participant prior to all measurements. Participants are collected from Shanghai Mental Health Center, including 54 people in the control group, 41 people with anxiety disorder, 39 people with bipolar disorder, 66 people with depression disorder, 5 people with obsessive–compulsive disorder, and 33 people with schizophrenia.

## Result and discussion

**Figure 3:**
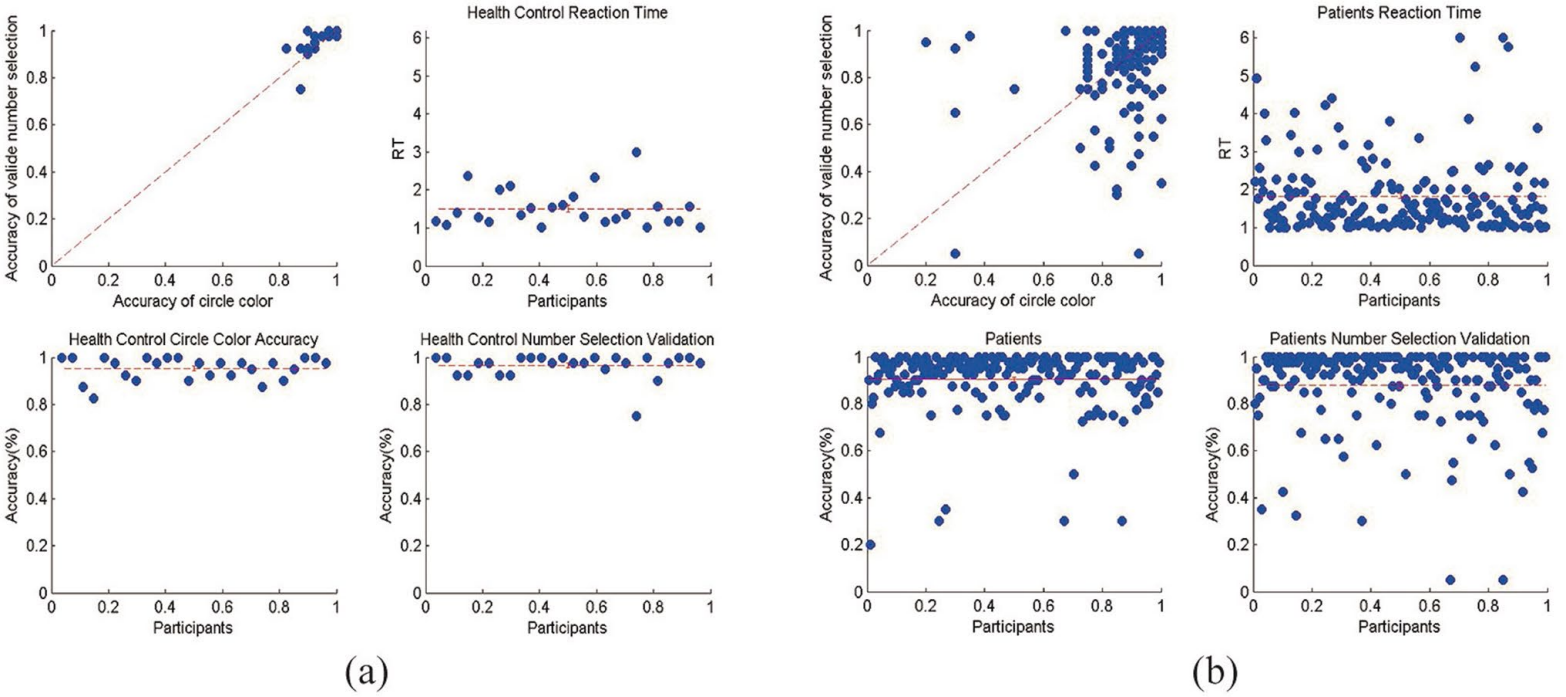
Result of attention test. (**a**) Accuracy and response results of control group; (**b**) accuracy and response results of mental disorder patients.

We selected four representative test results for analysis and discussion among 16 different cognitive ability-related tests in this system. Here is an example of the test that do not require to record any behavioral and physiological data that used external module. we obtained the attention test results between 92 people with mental disorders and 26 control groups. Based on the original data, we extracted the reaction time, the accuracy of the color of the circle and the accuracy of the corresponding number that selected. As shown in Fig. 3, the response time and accuracy of the patient group were significantly lower than those of the control group (P < 0.05).

**Figure 4:**
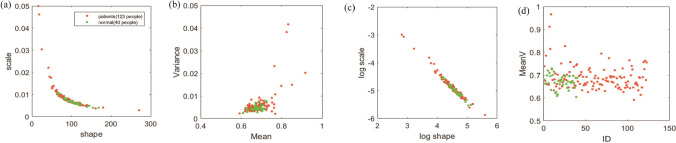
Hand movement test. (**a**) The shape and scale parameter of the maximum value of the motion speed Gamma distribution in the 95% confidence interval. The vertical axis can also be regarded as the Fano factor of the distribution. (**b**) The mean and variance of the Gamma distribution. (**c**) The shape parameter and scale parameter after log–log transformation. (**d**) Scatter plot of the average speed of each subject's exercise.

In the hand movement test that records hand tracking data, we obtained data samples of 40 control group and 123 schizophrenia patients. The results of their hand tracking data analysis are shown in Fig. 4, where the green dots are the control group. The red dots are patients with schizophrenia. As shown in Fig. 4a, we use the maximum likelihood estimation to find the shape and scale parameters of the Gamma distribution with the maximum value of the motion speed in the 95% confidence interval to measure the variability of the maximum value of the subject’s motion speed, among which, The larger the scale parameter, the greater the noise of the subjects’ kinematics parameters. In addition, we also calculated their average speed. As shown in Fig. 4b,d, the average speed of the control group is relatively concentrated, while the variance of the patient group is larger.

**Figure 5:**
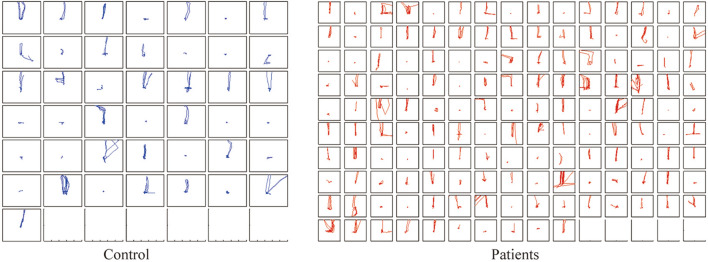
Results of eye tracking between patients and control group.

In the eye tracking test, we have recorded the changes in the subjects’ eye movements. We obtained the eye tracking test data of 145 patients with mental disorders and 43 people of control group. The eye movement trajectory data where the target appears on the top is shown in Fig. 5. Although the eye movement error of mental disorder patients is greater than control group, the difference in eye movement trajectories between healthy people and patients is not obvious. It was also found that the psychiatric patients and the control group showed no significant differences in eye saccade trajectories in the rest of the individual tasks. However, patients with mental disorders in tasks such as reaction time and reverse saccades showed obvious differences from the control group. This may be due to the fact that the eye trajectory of the patients in this experiment performed normal behavior in the saccade task, but in other tests, such as free viewing of pictures, the eye trajectory parameters are still important variables.

A three-lead portable EEG acquisition device was used to record resting EEG and EEG under music stimuli from normal people and patients, here we selected 9 people with anxiety, 10 people with bipolar disorder, 21 people with depression, 9 people with schizophrenia, and 9 people with schizophrenia and 32 healthy controls. We segmented the EEG data that under music stimuli into several parts, and then extracted 3 linear features and 3 non-linear features these different parts of EEG data, and then used SVM classifier to establish an optimization model by leaving one subject cross-validation method, and then used the test data to test the best Optimize the model to obtain the classification accuracy rate, the accuracy rate is shown in Table 4. Among them, the resting state EEG characteristics of schizophrenia are more obvious, and the classification accuracy can reach 89.02%. Under the music stimulation environment, the overall classification accuracy of the 4 types of symptoms were maintained at about 83%. Although music stimulation will induce changes in the patient's emotional characteristics and other psychological characteristics, and we hope that the stimulation can improve the recognition in the resting state, but it can be seen from the results that the classification accuracy of schizophrenia has been reduced. This may be due to the characteristics of the EEG under task induction did not enhance the difference between patients with mental disorders and the control group. For the patients with schizophrenia in the experiment, music stimulation made their EEG characteristics more consistent with those of the control group.

**Table 4:** Classification of mental disorders based on EEG.

Mental disorder	Accuracy under state EEG (%)	Accuracy under music stimuli (%)
Anxiety	85.37	85.29
Bipolar affective disorder	82.14	82.14
Depression	80.19	83.33
Schizophrenia	89.02	82.86

The various behavioral and physiological data collected in the proposed test bed can also be used for other types of cognition research. For example, eye movement behavior detection in the system is often used in reading habits and attention preferences. The EEG of the prefrontal lobe also has broad application prospects in attention monitoring, event-related potential research, sleep status and other cognitive-related fields. Minor changes in hands or other physical behaviors are also significant behavioral indicators in a variety of abnormal behavior research. Therefore, this system has universal applicability and can study a variety of cognitive states and abilities by combining different experimental paradigms. In the application examples of mental disorders used in this article, we hope to enrich the diagnosis and restorative testing of mental disorder through the assessment of cognitive ability. Therefore, in the process of diagnosing mental disorder, the proposed system provides multiple indicators such as interactive behavior, eye movement behavior and EEG in cognitive tasks, and these indicators also enrich the symptom performance of some patients. According to The World Health Organization Disability Assessment Schedule^45^, the patient is assessed to perform activities in six areas: understanding and communicating; getting around; self-care; getting along with people; life activities; and participation in society. These factors are basically included in the theoretical framework of cognitive assessment. In the assessment of cognitive and behavioral ability of patients with mental disorders, schizophrenic patients may be affected by hallucinations, abnormal behaviors and other factors, so that their cognitive ability in a certain aspect will be greatly reduced or increased^46^. Depression may be accompanied by cognitive changes, so the test can be used for long-term evaluation of the depression group. Anxiety disorders include disorders that share features of excessive fear and anxiety and related behavioral disturbances. ASD is diagnosed when the characteristic deficits of social communication are accompanied by excessively repetitive behaviors, restricted interests, and insistence on sameness, and we hope to obtain more quantitative indicators of cognitive differences in different types of ASD disorders. Therefore, the main purpose of this system is to supplement and enhance the accuracy of the diagnostic process during multiple behavioral and physiological characters.

We designed a specific cognitive ability evaluation paradigm for each indicator, based on the traditional cognitive assessment paradigm and the acceptance of patients with mental disorders. It can be found that the difficulty of each paradigm is slightly different among those tests. It can be found that the difficulty of executing each paradigm is slightly different. Considering that patients with symptoms such as ASD may exceed ordinary people in a certain cognitive ability, such as memory, biometric recognition, hearing tests, etc. For those tests, people do not suffer from any mental disorders may have some errors during the test. Some tests, such as assessment of attention, cognitive control, and Anti saccade, are clearly designed for patients with mental disorders. Therefore, we hope that this integrated cognitive ability assessment battery can cover all basic cognitive abilities. In fact, whether it is based on the evaluation scope of neurocognitive disorders or on the basic theoretical framework of cognitive assessment, those 16 tests we provided basically cover all dimensions of cognitive ability. In addition, the purpose of this system is to provide some objective behavioral indicators to the doctor, and clinical doctors still need to combine traditional manuals, scales, observations, and medical histories to diagnose patients. The system we designed can only be used to further track and study the cognitive abilities of patients after they are diagnosed. In addition, it will provide effective support for personalized and quantitative medical big data in the future.

In fact, our system is to integrate multiple cognitive assessment methods, EEG recording, eye tracking, and motion tracking devices. The high level of integration is also the greatest advantage of this system. Due to the integrated design of multiple experiments, it has high test consistency and does not suffer from measurement errors caused by systematic delays resulting from different experiments. Additionally, it is more time-efficient when using this system for cognitive-behavioral screening of mental disorders. The experimenter can screen as many different cognitive behaviors as possible in a single system, making it more convenient to associate cognitive and mental behaviors with diseases. Currently, the analysis programs for corresponding experiments have not been added, and only raw data collection is provided. This is mainly due to the lack of unified data analysis methods for different behavioral experiments. In next work, it will be necessary to increase data volume and functionality iterations to integrate universal analysis programs into this system.

In the future research, we may replace the low-correlative and unsuitable cognitive assessment paradigm and add new cognitive assessment methods with better discriminative characters in the research. In addition, we will also add other new behavior indicators based on diagnosis needs, such as adding real-time information such as myoelectricity and cerebral blood oxygen, or adding a facial expression-based emotion recognition system. In the existing evaluation paradigm, we currently have fixed relevant parameters in the system, such as the memory time in memory evaluation, the duration of graphics and numbers in the attention test, the number of trails, etc. In the future, the parameters in the system can also be set to be changeable, to be more suitable for actual testing. In terms of data management, this system collects various types of experimental data, including basic parameters and result data of each experiment, eye tracking data, hand moving data, EEG data, audio data, etc. Their file formats and sizes are different, so metadata (data describing data, used to describe the characteristics and attributes of data) can be further used to manage and store these data. For example, the distributed file system HDFS can be used to effectively use metadata to manage data of various formats and sizes^47^. Using Hadoop can also compress and store big data such as EEG, manual, and video, saving server resources^48^. Using metadata to store the raw data of each cognitive evaluation paradigm also helps other researchers to effectively process the data through experimental parameters and details. Now the system is implemented separately (client and web-based version), and a unified platform is needed for the integration of all functions. After the integration, part of the database can be made open source and available for researchers from all over the world.

## Conclusions

This research mainly introduced a multi-source cognitive assessment test-bed for behavioral and physiological data measurement, which can record multi-pattern feedback at different spatiotemporal levels simultaneously. Under this test-bed, a diagnostic toolset for mental disorders is developed, which integrated 16 cognitive assessment test tools including attention, working memory, cognitive control, decision-making and judgment, etc. The hardware platform can record behavioral and physiological data, including recording 3-leads EEG, hand movement, eye tracking and voice, the software platform can be visited from internet or operated locally. The controlled experiments show this diagnostic toolset supports for screening of mental disorder behavioral indicators and individualized medical big data analysis.

## Data Availability

The datasets generated during the current study are not publicly. Because of the protection policy of behavioral and physiological data in the Institute of Automation. But are available from the corresponding author on reasonable request.
